# Management of acute aortic syndrome with evolving individualized precision medicine solutions: Lessons learned over two decades and literature review

**DOI:** 10.3389/fsurg.2023.1157457

**Published:** 2023-03-28

**Authors:** Sherif Sultan, Yogesh Acharya, Keegan Chua Vi Long, Mohamed Hatem, Mohieldin Hezima, David Veerasingham, Osama Soliman, Niamh Hynes

**Affiliations:** ^1^ Western Vascular Institute, Department of Vascular and Endovascular Surgery, University Hospital Galway, University of Galway, Galway, Ireland; ^2^ Department of Vascular Surgery and Endovascular Surgery, Galway Clinic, Royal College of Surgeons in Ireland and University of Galway, Galway Affiliated Hospital, Doughiska, Ireland; ^3^ CORRIB-CURAM-Vascular Group, University of Galway, Galway, Ireland; ^4^ Department of Cardiothoracic Surgery, University Hospital Galway, University of Galway, Galway, Ireland

**Keywords:** acute aortic syndrome (AAS), thoraco-abdominal aorta, thoracic endovascular aneurysm repair (TEVAR), hybrid endovascular repair, stent-graft induced new entry tear (SINE)

## Abstract

**Background:**

Thoracoabdominal acute aortic syndrome is associated with high morbidity and mortality. We aim to scrutinize our evolving strategies for acute aortic syndrome (AAS) management using minimally invasive and adaptive surgical techniques over two decades.

**Methods:**

This is a longitudinal observational study at our tertiary vascular centre from 2002 to 2021. Out of 22,349 aortic referrals, we performed 1,555 aortic interventions over twenty years. Amongst 96 presented with symptomatic aortic thoracic pathology, 71 patients had AAS. Our primary endpoint is combined aneurysm-related and cardiovascular-related mortality.

**Results:**

There were 43 males and 28 females (5 Traumatic Aortic Transection (TAT), 8 Acute Aortic Intramural Hematoma (IMH), 27 Symptomatic Aortic Dissection (SAD) and 31 Thoracic Aortic Aneurysm (TAA) post-SAD) with a mean age of 69. All the patients with AAS received optimal medical therapy (OMT), but TAT patients underwent emergency thoracic endovascular aortic repair (TEVAR). Fifty-eight patients had an aortic dissection, of which 31 developed TAA. These 31 patients with SAD and TAA received OMT initially and interval surgical intervention with TEVAR or sTaged hybrId sinGle lumEn Reconstruction (TIGER). To increase our landing area, we performed a left subclavian chimney graft with TEVAR in twelve patients. The average follow-up duration was 78.2 months, and eleven patients (15.5%) had combined aneurysm and cardiovascular-related mortality. Twenty-six percentage of the patients developed endoleaks (EL), of which 15% required re-intervention for type II and III. Four patients who had paraplegia (5.7%) and developed renal failure died. None of our patients had a stroke or bowel ischaemia. Twenty patients had OMT, eight of these were patients with acute aortic hematoma, and all eight died within 30 days of presentation.

**Conclusion:**

Acute aortic hematoma is a sinister finding, which must be closely monitored, and consideration is given to early intervention. Paraplegia and renal failure result in an increased mortality rate. TIGER technique with interval TEVAR has salvaged complex situations in young patients. Left subclavian chimney increases our landing area and abolishes SINE. Our experience shows that minimally invasive techniques could be a viable option for AAS.

## Introduction

Acute pathologies of the thoracoabdominal aorta involve life-threatening aortic conditions associated with high mortality rates (1–4). The true incidence of these pathologies is unknown, but it is estimated that aneurysms originating in the descending thoracic aorta occur at an incidence of 5.9–10.4 per 100,000 person-year and rupture at a rate of 3.5 per 100,000 person-year (1, 2, 4). These disorders are more common in the elderly, and their prevalence increases with age.

Bickerstaff et al. (5), in a population-based study of patients with untreated thoracic aneurysms, reported 5-year survival rates of 19.2%, with 51% of the deaths attributed to aneurysm rupture. A prohibitive high clinical index is mandatory to detect the pathology in patients with intractable chest and interscapular pain so that appropriate intervention can be undertaken to reduce loss of life and devastating life-altering complications (6).

Thoracic endografting has emerged as an effective alternative treatment for thoracic aortic pathology over the last three decades; however, its role in managing other complex aortic pathologies is yet to be established. The European Society of Cardiology (7) has a comprehensive flowchart for decision-making based on pre-test sensitivity. Such management algorithms may help to diminish the diagnostic delay in emergency scenarios. However, generalised approaches can sometimes fail to appreciate subtle nuances between individual patients and disease presentations; therefore, a pre-emptive personalised approach is often necessary for the optimal management of acute aortic syndrome (AAS) with relatively different contributions of surgical repair, endovascular intervention and optimal medical therapy (OMT).

## Methods

This longitudinal observational study analysed patient demographics, the nature of aortic pathology, mode of intervention and clinical outcome and scrutinised our experience managing AAS at a vascular tertiary referral centre.

We performed 1,555 aortic interventions amongst 22,349 aortic referrals over twenty years (2001–2021). Amongst them, 96 presented with symptomatic aortic thoracic pathology and 71 had AAS.

### Acute aortic syndrome (AAS)

An established diagnosis of AAS was confirmed if the thoracic aorta was complicated by rupture, hematoma, dissection, mal-perfusion syndrome, and acute aortic expansion, as defined by the European Society of Cardiology (ESC) (8) and the Society for Vascular Surgery/Society of Thoracic Surgeons (SVS/STS) (9) guidelines. We followed reporting standards (8, 9) nomenclature to classify AAS in order to provide a granular description of our study populations and the AAS disease processes.

For patients with a high index of AAS suspicion, a rapid, comprehensive diagnostic workup was performed, including clinical assessment, D-Dimer and troponin, electrocardiogram (ECG) and ECG-gated computed tomography angiography (CTA) aortic angiogram, which included the great head and neck vessels, visceral, and pelvic arterial supply.

To limit the extension of the dissection and reduce the risk of developing end-organ damage and rupture, all of our AAS patients, regardless of definitive therapeutic interventions, had initial medical therapy to decrease wall stress (10). First, we introduce adequate control of pain by intravenous opiate analgesia; then we aim at heart rate <60 b.p.m and systolic blood pressure (SBP) between 100 and 120 mmHg in a high dependency unit (HDU) or intensive care unit (ICU) setting (8). Intravenous labetalol and sodium nitroprusside are tailored to rapidly achieve optimal blood pressure levels. However, in patients who are intolerant to beta-blockers and do not suffer from vascular connective tissue disorder, we supplement with non-dihydropyridine calcium channel antagonists (verapamil and diltiazem).

### Types of interventions

We used endovascular intervention either with thoracic endovascular aortic repair (TEVAR) or sTaged hybrId sinGle lumEn Reconstruction (TIGER) techniques (11).

TIGER technique includes staged open and endovascular intervention to form a uni-lumen aorta, starting with an open surgery from the supra celiac—infra-diaphragmatic aorta to bilateral common iliac arteries, contemplated with aorto-visceral endarterectomies with “Silver Dacron” surgical patching (11). Three months later, we performed proximal endovascular intervention with TEVAR to deal with inflow pathology.

All devices used were commercially available and conformed to CE-Marked (Communauté Européenne) standards. Devices were used according to manufacturers' instructions except when using chimney grafts. Thoracic endografts used included the cTAG (Gore Medical, Flagstaff, AZ, United States), Talent (Medtronic, Santa Rosa, CA, United States), Valiant stents (Medtronic, Santa Rosa, CA, United States), Streamlinears flow modulators (SL) (Cardiatis, Isnes, Belgium) and Inner Branch (Artivion, NW Kennesaw, GA 30144, United States). The Bentley and Bentley plus (Bentley InnoMed GmbH, Hechingen, Germany), and Viabahn endoprosthesis (Gore Medical, Flagstaff, AZ, United States) were used as a chimney or branched endovascular aneurysm repair (BEVAR) grafts. The abdominal debranching and re-anastomosis were carried out using an integrated knitted quadrifurcated graft (Maquet GmbH & Co. KG, Rastatt, Germany).

### Outcome measures and endpoints

The primary endpoint was a combined aneurysm and cardiovascular related-mortality. A combined aneurysm and cardiovascular-related mortality were chosen because thoracic aneurysm repair induces adaptive left ventricular hypertrophy with non-occlusive myocardial ischaemia with diastolic dysfunction regardless of the intervention types (12, 13). Secondary endpoints included technical success, freedom from reintervention, and all-cause mortality.

### Perioperative patient management

Patients were routinely followed in an ICU for a minimum of 24 h. Spinal drainage was employed in our early experience; for the past eight years, we do not routinely do spinal drainage as all of our cases are performed as a staged approach. Spinal drainage was not used at any stage in patients undergoing TIGER or the first stage of debranching.

Hydration was administered before and after procedures in all patients regardless of renal profile to prevent the development of contrast nephropathy, which is a preventable complication that, in emergencies, could add to the already existing threat of acute tubular necrosis from malperfusion or hypotension. Systemic intra-operative heparinization was used, maintaining patients activated partial thromboplastin time (aPTT) between 60 and 80 s. Intraoperative heparinization was monitored using lab aPTT and theatre activated clotting time (ACT) to get instantaneous results. Until 2012, we used adenosine to arrest the heart before the deployment of our stents. Currently, we use temporary pacing to fibrillate the heart to decrease the cardiac out. All patients with traumatic transection had an occluding inferior vena cave balloon to decrease the physiological cardiac output.

### Follow-up

Patients were followed by clinical investigation, ankle-brachial indices (ABI), duplex ultrasound (DUS) of the abdominal aorta and subclavian artery, plain film x-ray and blood sample tests before discharge, at six weeks, and six-monthly intervals for 24 months and yearly after that. Where CTAs were performed, Digital Imaging and Communications in Medicine (DICOM) images were analysed using 3-mensio reconstruction software (3-mensio Medical Imaging BV Bilthoven, The Netherlands).

### Statistics

Data, mainly collected from electronic patient records, was also gathered from the prospectively updated Vascubase data system (Consensus Medical Systems Inc, Richmond, Canada), which provided information on the stents used, procedure types and complications. The institutional review board approved the study.

Data collected from the prospectively updated database was analysed using Jamovi (Version 2.3, Jamovi project 2022, Sydney, Australia). Continuous variables are described as mean ± SD, and comparisons between groups are made using the *t*-test. Cumulative all-cause and aneurysm-related survival rates were estimated using Kaplan-Meier analysis, and curves were compared using the Log-rank test. Statistically significant differences were considered at *p* < 0.05.

## Results

We had 71 patients with AAS (5 Traumatic Aortic Transection (TAT), 8 Acute Aortic Intramural Hematoma (IMH), 27 Symptomatic Aortic Dissection (SAD) and 31 Thoracic Aortic Aneurysm (TAA) post-SAD). The baseline characteristics of these patients are given in Table 1.

**Table 1:** Baseline demographics of the patients.

Baseline characteristics	Acute Aortic Syndrome (AAS) (*N* = 71)					
	Overall	Types				
		TAT (*N* = 5)	IMH (*N* = 8)	SAD (*N* = 27)	TAA (Post-SAD) (*N* = 31)	*p*-value
**Total**	71	7.04%	11.26%	38.02%	43.66%	–
**Male, *n***	43	3	2	20	18	–
**Age (Mean ± SD), years**	69.2 ± 14.7	52.7 ± 23.3	77.5 ± 11.8	66.6 ± 15.5	72.0 ± 10.6	0.087
**Hypertension**	59	1	7	25	26	0.001*
**Hypercholesterolimia**	15	1	2	3	9	0.413
**Cardiovascular Disease**	28	0	4	9	15	0.165
**Diabetes Mellitus**	7	0	0	3	4	0.617
**Stroke**	5	0	2	1	2	0.187
**Smoker**	35	2	3	14	16	0.860

TAT, traumatic aortic transection; IMH, intra mural hematoma; SAD, symptomatic aortic dissection; TAA, thoracic aortic aneurysm; SD, standard deviation.

All the patients with IMH received OMT, and all the patients with TAT underwent emergency TEVAR (Figure 1). Fifty-eight patients presented with aortic dissection, and all received OMT, of which 31 developed symptomatic TAA (Figures 2–6) on an average of four years post aortic dissection (TAA post-SAD) and required intervention; the remaining cohort underwent interval surgical intervention with TEVAR or TIGER (Figures 7–11).

**Figure 1:**
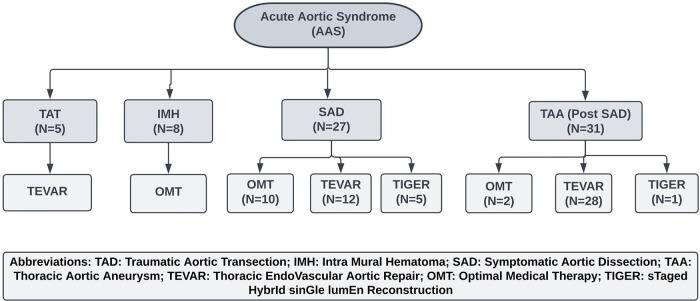
Management of acute aortic syndrome. OMT, Optimal Medical Therapy; TEVAR, Thoracic Endovascular Aneurysm repair; TIGER, STaged HybrId sinGle lumEn Reconstruction.

**Figure 2:**
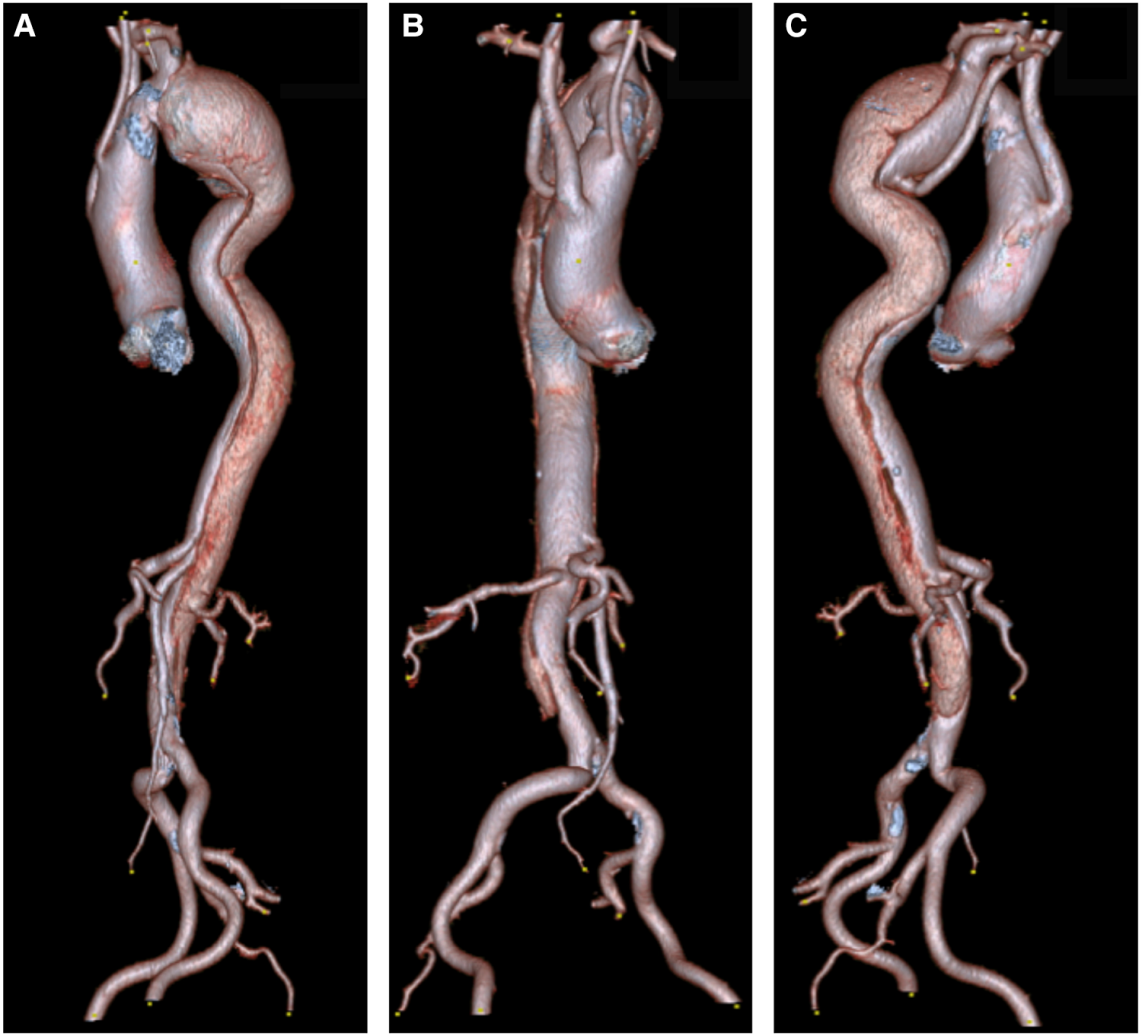
(**A**) A 3D reconstruction of type B aortic dissection, starting from the origin of the left subclavian to the distal aorta. (**B,C**) Depicts an aberrant right subclavian artery that arises from the mid-descending aorta. Note that both common carotids arise from ascending aorta.

**Figure 3:**
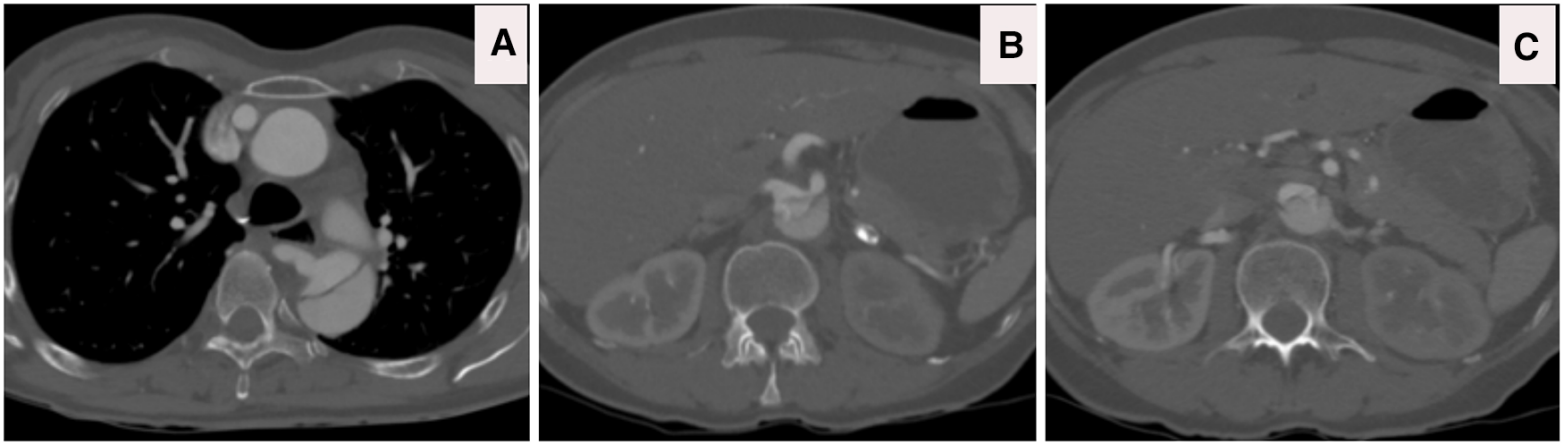
(**A**) CTA axial view illustrates that the aberrant right subclavian artery arises from the compressed true lumen. (**B**) CTA axial view confirms complex dissection at the level of celiac, superior mesenteric artery (SMA) and right renal artery with dynamic and static occlusion. (**C**) CTA axial view confirms complex dissection at the level left renal artery with dynamic and static occlusion, with main blood supply arising from false lumen with the hypoperfused left kidney.

**Figure 4:**
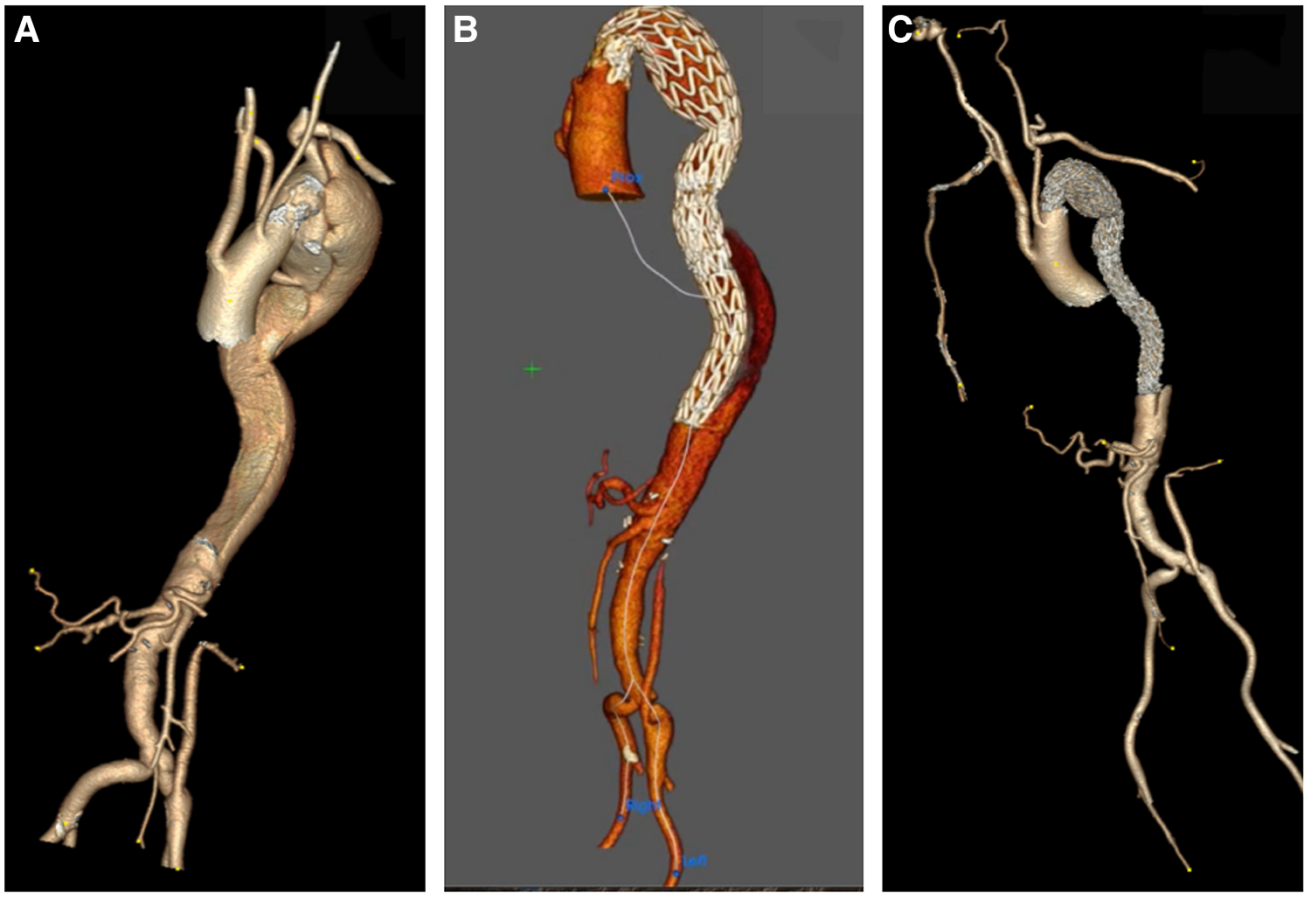
(**A**) 3D reconstruction post sTaged hybrId sinGle lumEn reconstruction (TIGER), demonstrating patient all visceral with left common iliac to left renal artery bypass. (**B,C**) A 3D reconstruction post-TIGER and thoracic endovascular aortic repair (TEVAR) with Gore C-TAG, with bilateral transposition of both subclavian arteries to both common carotid, demonstrating patent all great vessels of head and neck and all visceral vessels.

**Figure 5:**
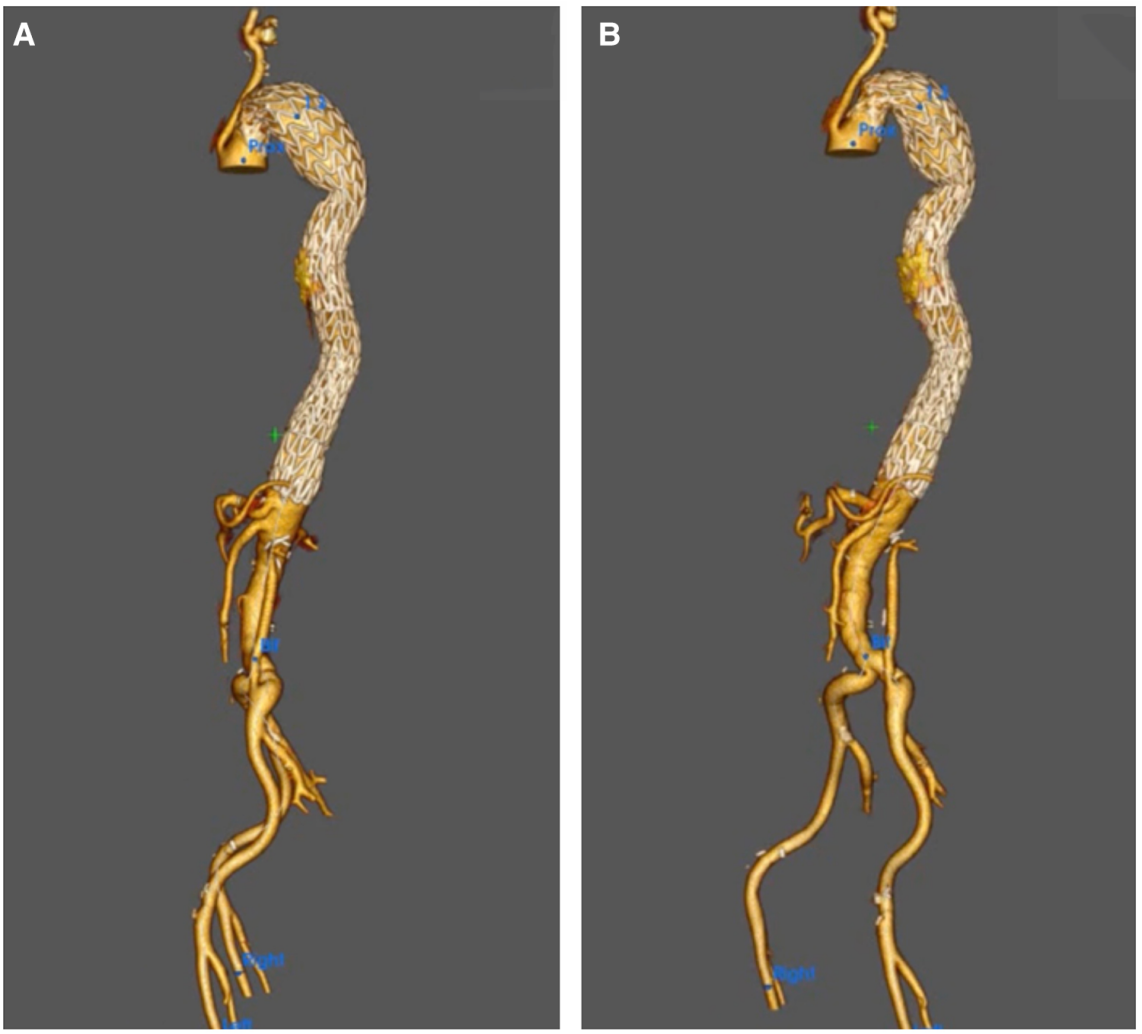
(**A,B**) A 3D reconstruction post-knickerbocker technique to seal distal thoracic aortic aneurysm (TAA) and abolish type IB with gore C-TAG, demonstrating total modulation of TAA.

**Figure 6:**
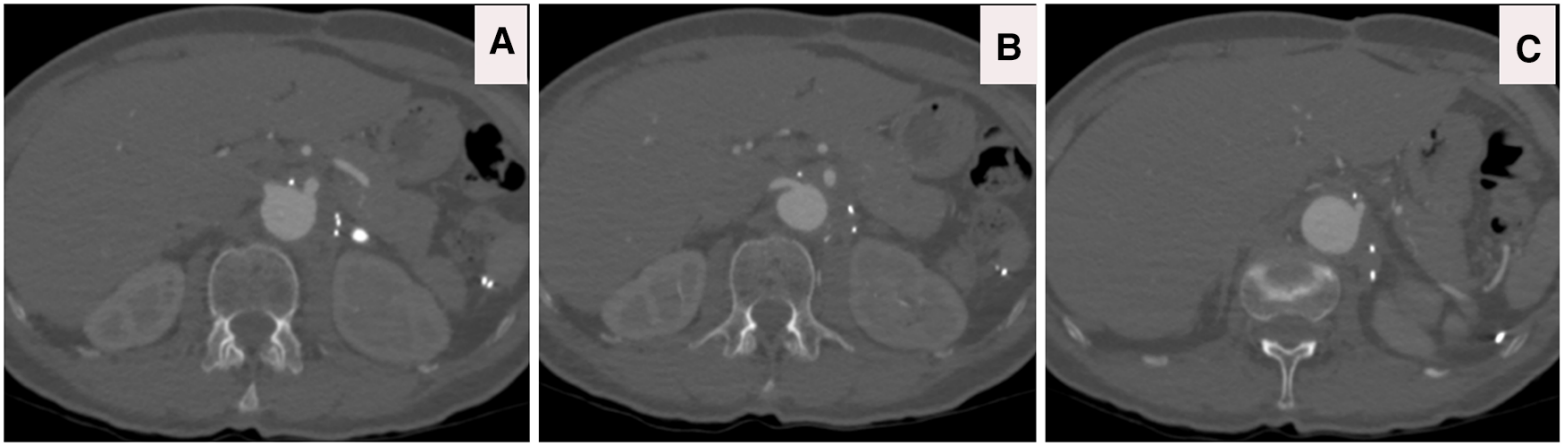
(**A–C**) CTA axial views four years post sTaged hybrId sinGle lumEn reconstruction (TIGER) without any evidence of aneurysmal diseases or dissection or stenosis; patients are off all of her antihypertensive medications.

**Figure 7:**
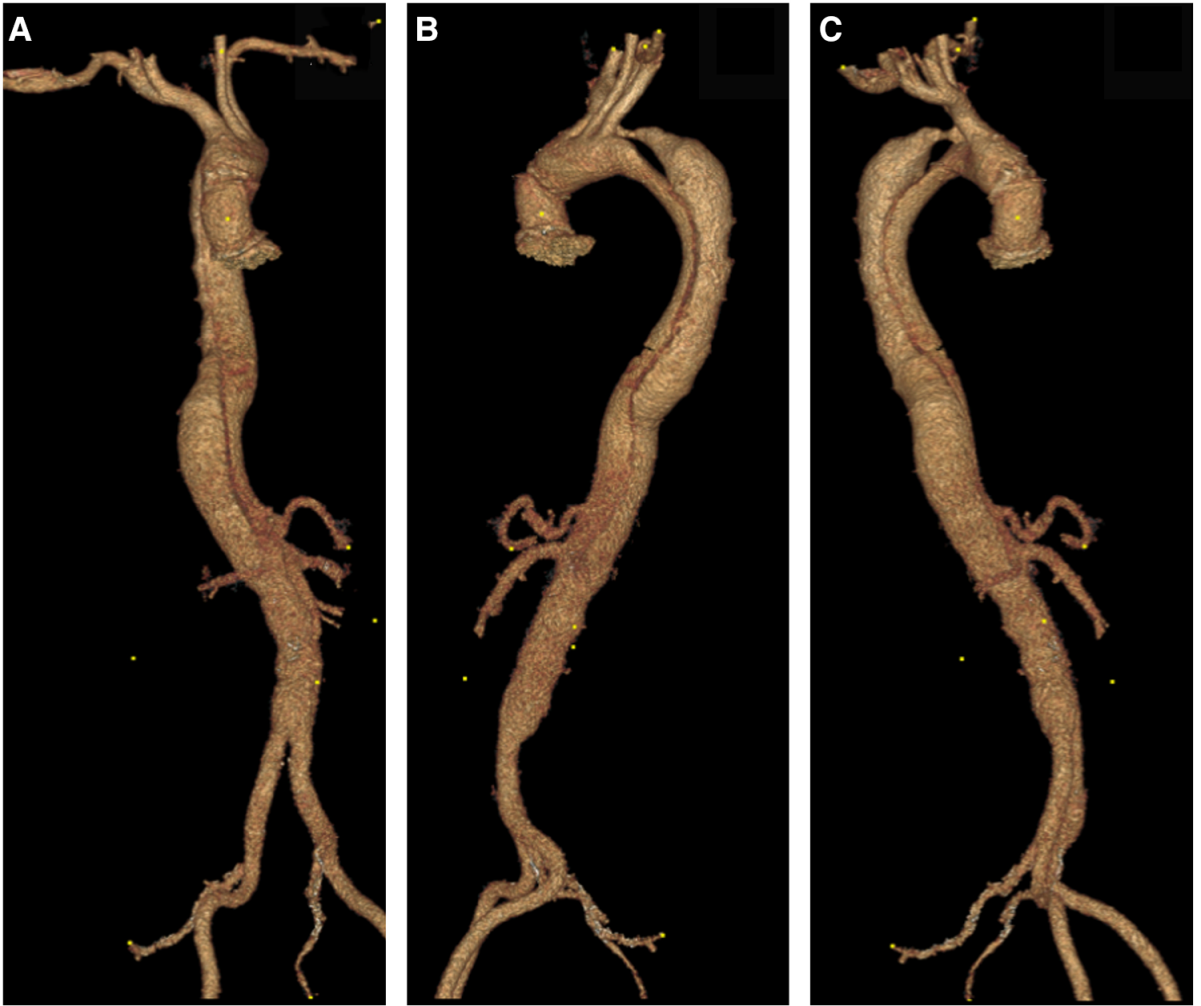
(**A–C**) Day one post type A aortic dissection, 3D reconstruction confirms that the dissection involves the left subclavian artery and extends to the mid-infra renal aorta, celiac axis, superior mesenteric artery (SMA) and left renal arises from false lumen with hypoperfusion.

**Figure 8:**
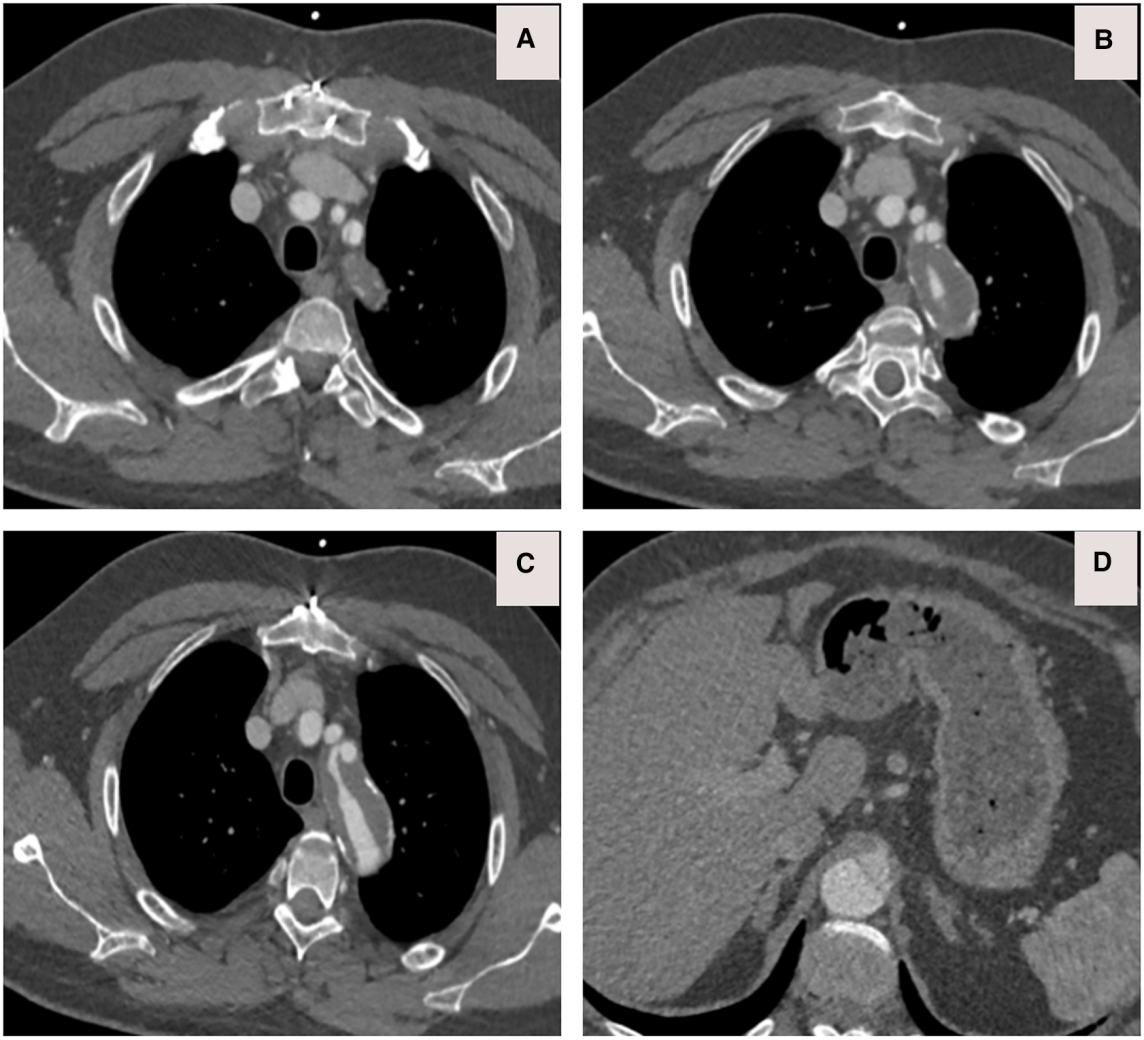
(**A**) CTA axial view depicts that the dissection extends to the left subclavian artery. (**B,C**) CTA axial views confirm the ominous sign of acute false lumen thrombosis that supplies visceral arteries. (**D**) CTA Axial view demonstrates that the celiac axis arises from a false lumen.

**Figure 9:**
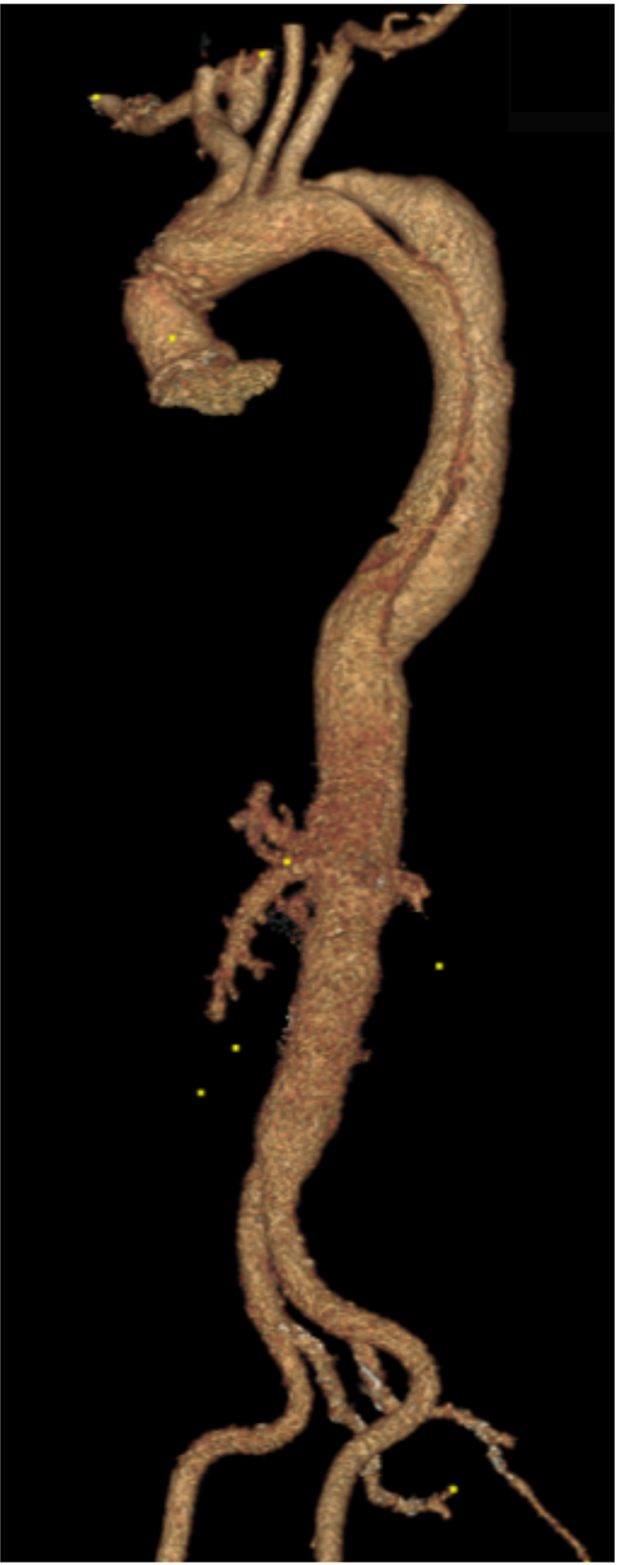
A 3D reconstruction post sTaged hybrId sinGle lumEn reconstruction (TIGER) demonstrating well-perfused visceral organs.

**Figure 10:**
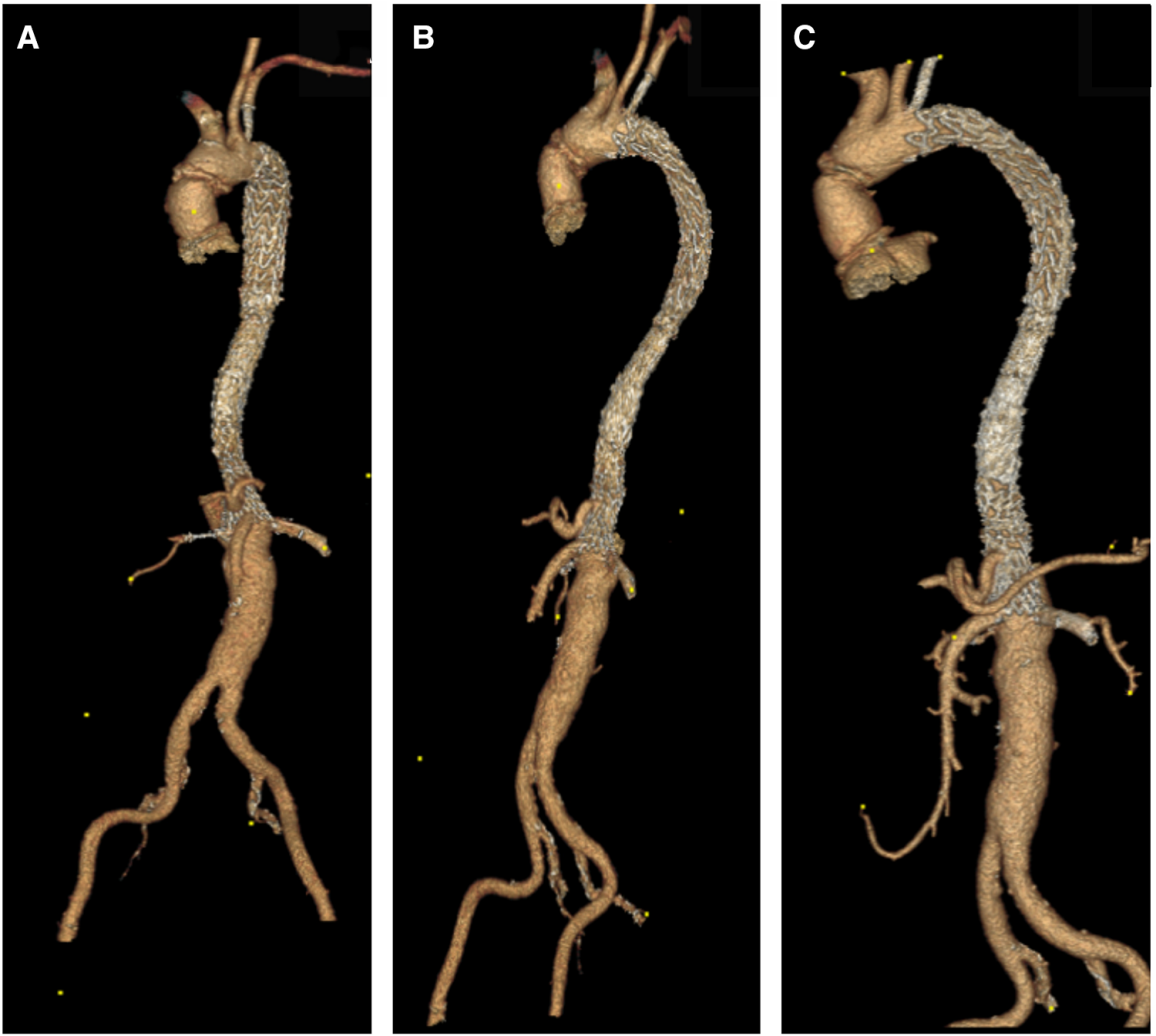
(**A–C**) A 3D reconstruction post sTaged hybrId sinGle lumEn reconstruction (TIGER) and thoracic endovascular aortic repair (TEVAR) with a chimney to the left subclavian artery confirming total modulation of the aorta.

**Figure 11:**
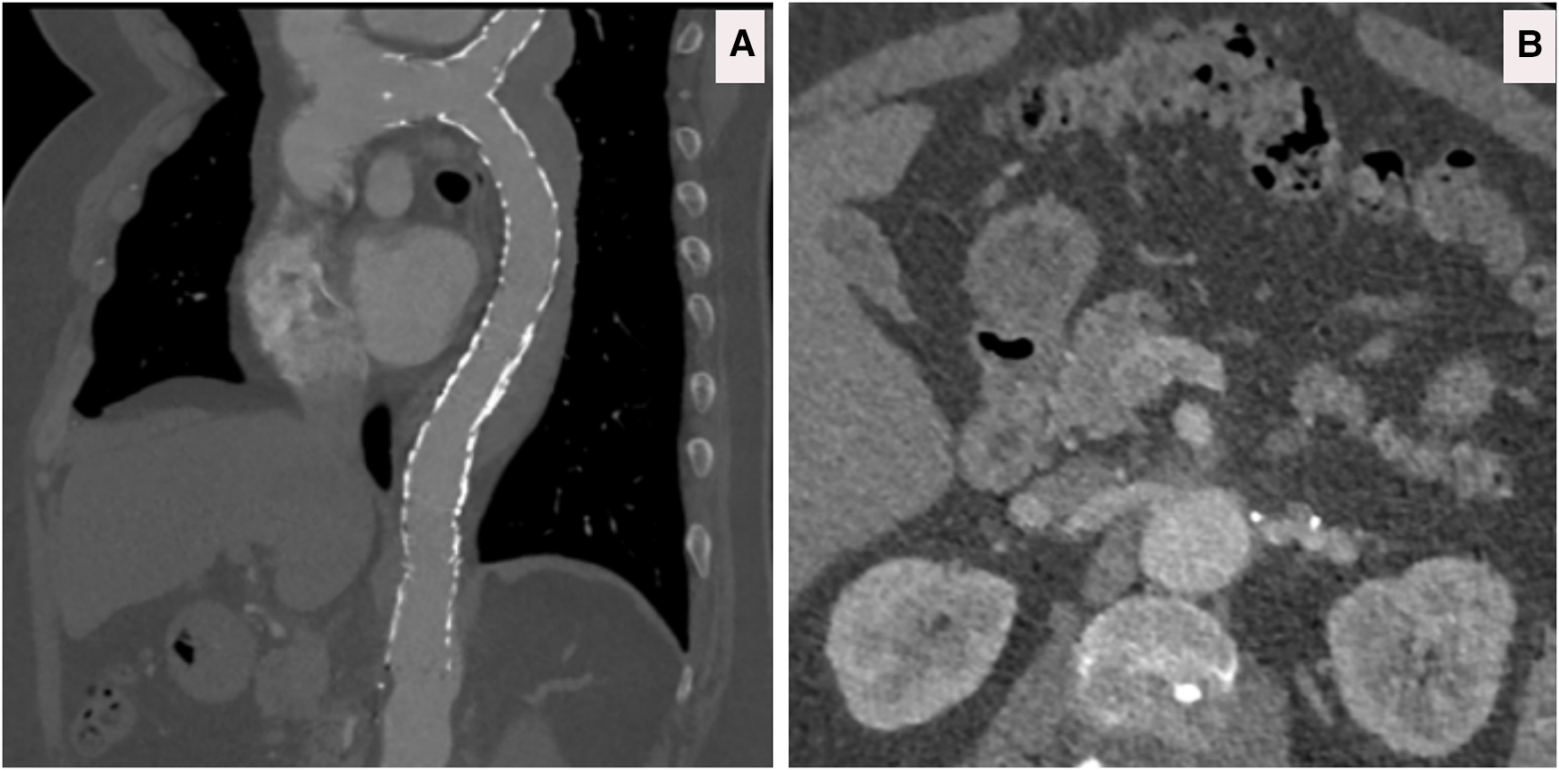
(**A**) CTA sagittal views confirming the patency of the left subclavian chimney and the thoracic endovascular aortic repair (TEVAR) gore C-TAG graft. (**B**) CTA axial views, five years post sTaged hybrId sinGle lumEn Reconstruction (TIGER), show well-perfused kidneys without any evidence of dissection, aneurysmal dilatation or restenosis.

Twelve patients had left subclavian chimney graft along with TEVAR (1 TAT, 2 SAD, 9 TAA post-SAD) to increase the length of proximal landing zone into zone two and to reduce the risk of retrograde type A dissection and prevent Stent graft Induced New Entry tear (SINE). Eight cases of TEVAR were performed with transposition of the left subclavian artery to the left common carotid artery rather than utilising chimney stents because the length of the subclavian stents required would have impinged into the lumen of the left common carotid artery.

### Endograft types

Thirty-six Polytetrafluoroethylene (PTFE) and nitinol, 10 polyester (PE) and nitinol and five cobalt chromium-based endografts were used (Table 2). This included 36 Gore CTAG endoprosthesis (W. L. Gore & Assoc, Flagstaff, Ariz, United States) (5 TAT, 13 SAD, 18 TAA post-SAD), 10 Talent/Valiant (Medtronic Inc, Minneapolis, United States) (3 SAD and 7 TAA post-SAD), 5 Artivion BEVAR (Artivion, NW Kennesaw, GA 30144, United States) (1 SAD and 4 TAA post-SAD).

**Table 2:** Endograft types.

Stent Fabric Types	Total	Acute Aortic Syndrome (AAS)				
		TAT	SAD		TAA post-SAD	
		TEVAR	TEVAR	TIGER	TEVAR	TIGER
**PTFE Gore**	36	5	8	5	17	1
**Polyester Talent/Valiant**	10	0	3	0	7	0
**Artivion**	5	0	1	0	4	0

PTFE, polytetrafluoroethylene; PE, polyester; TAT, traumatic aortic transection; IMH, intra mural hematoma; SAD, symptomatic aortic dissection; TAA, thoracic aortic aneurysm; TEVAR, thoracic endovascular aortic repair; TIGER, sTaged hybrId sinGle lumEn Reconstruction.

Twelve patients (26%) developed endoleaks (EL), of which 7 (15%) required re-intervention for type I and III. Five Type 2 EL (3 SAD—1 TEVAR and 2 TIGER and 2 TAA post-SAD—following TEVAR), 6 Type 1 EL (2 SAD TEVAR and 4 TAA post-SAD following TEVAR), and 1 Type 3 EL (TAA post-SAD following TEVAR). Endoleak developed in 10 patients in whom PTFE-based devices were used (4 amongst patients with SAD with 1 Type 1 EL and 1 Type 2 EL following TEVAR and 2 Type 2 EL post-TIGER, and 6 TAA post-SAD with 4 Type 1 EL and 2 Type 2 EL following TEVAR) and in 2 patients in whom PE-based endografts were used (1 Type 1 EL in SAD following TEVAR and 1 Type 3 EL in TAA post-SAD following TEVAR). Secondary re-interventions were based on six type I ELs and one type I EL. Type I ELs required extensions proximally and distally, while type III EL underwent realignment in the middle of the graft.

There was 100% technical success for TEVAR and TIGER. All grafts were deployed proximally at zone 2 with the chimney, or at zone 3.

Seven patients with TEVAR (1 TAT, 2 SAD, 4 TAA post-SAD) died within 30 days of intervention. Four died from aneurysm rupture (1 SAD and 3 TAA post-SAD—“1 Gore, 1 Talent, 1 Valiant, 1 SL”), one systemic embolization (1 TAA Post-SAD—“SL”), thrombosis and compression, one stent-erosion into the bronchus and severe haemoptysis (TAA post-SAD—“Valiant”), and one organ failure secondary to DIC (TAD—“SL”).

**Figure 12:**
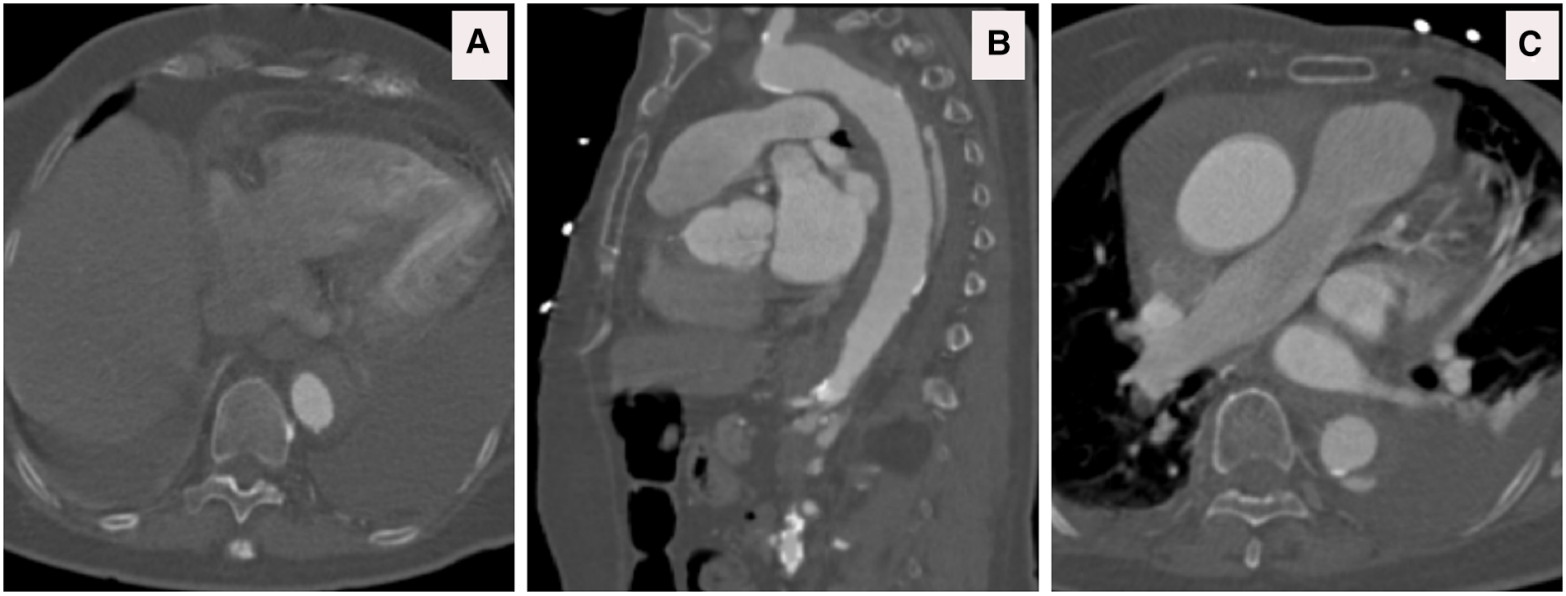
(**A**) CTA done at one-hour post-collapse in another institute shows acute aortic hematoma with interscapular pain during the COVID-19 pandemic lockdown. (**B**) CTA sagittal view done at 20 h post-collapse in another institute confirmed a small bleed into the acute mural hematoma. (**C**) CTA axial views demonstrated that the hematoma extends proximally and distally.

**Figure 13:**
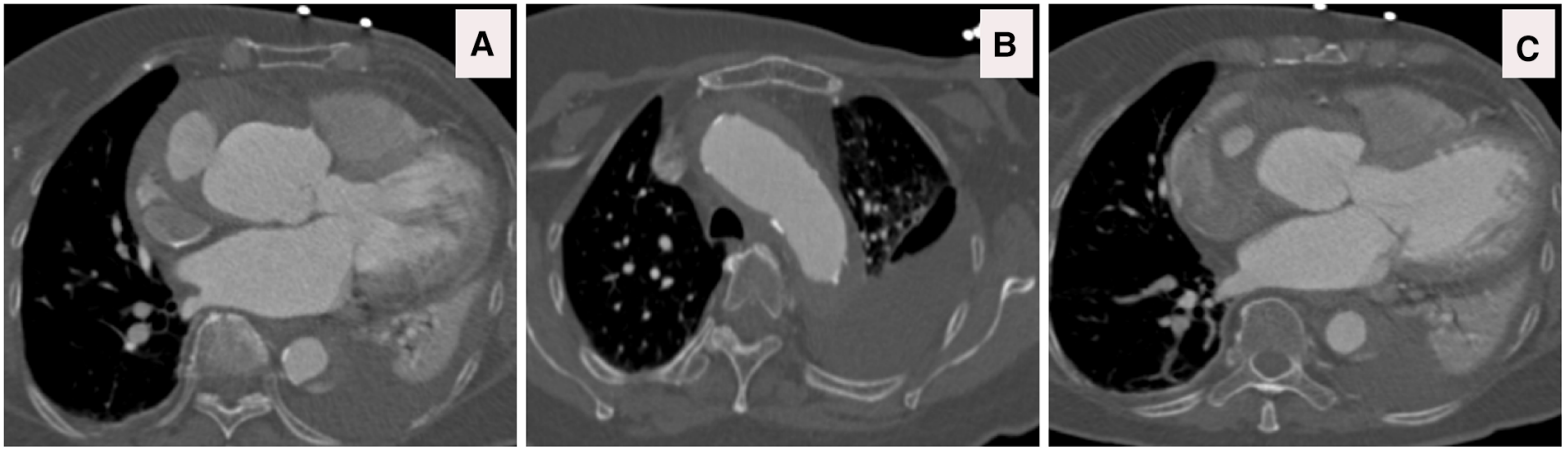
(**A–C**) CTA axial views demonstrated rupture of the ascending and descending aorta with acute catastrophic haemorrhage with a terminal event on patient arrival to our institute.

All eight acute aortic intramural hematomas died within 30 days of the diagnosis from rupture without undergoing any intervention as they were admitted initially under the referring team and were referred to us only on the occurrence of these catastrophic complications (Figures 12, 13).

**Figure 14:**
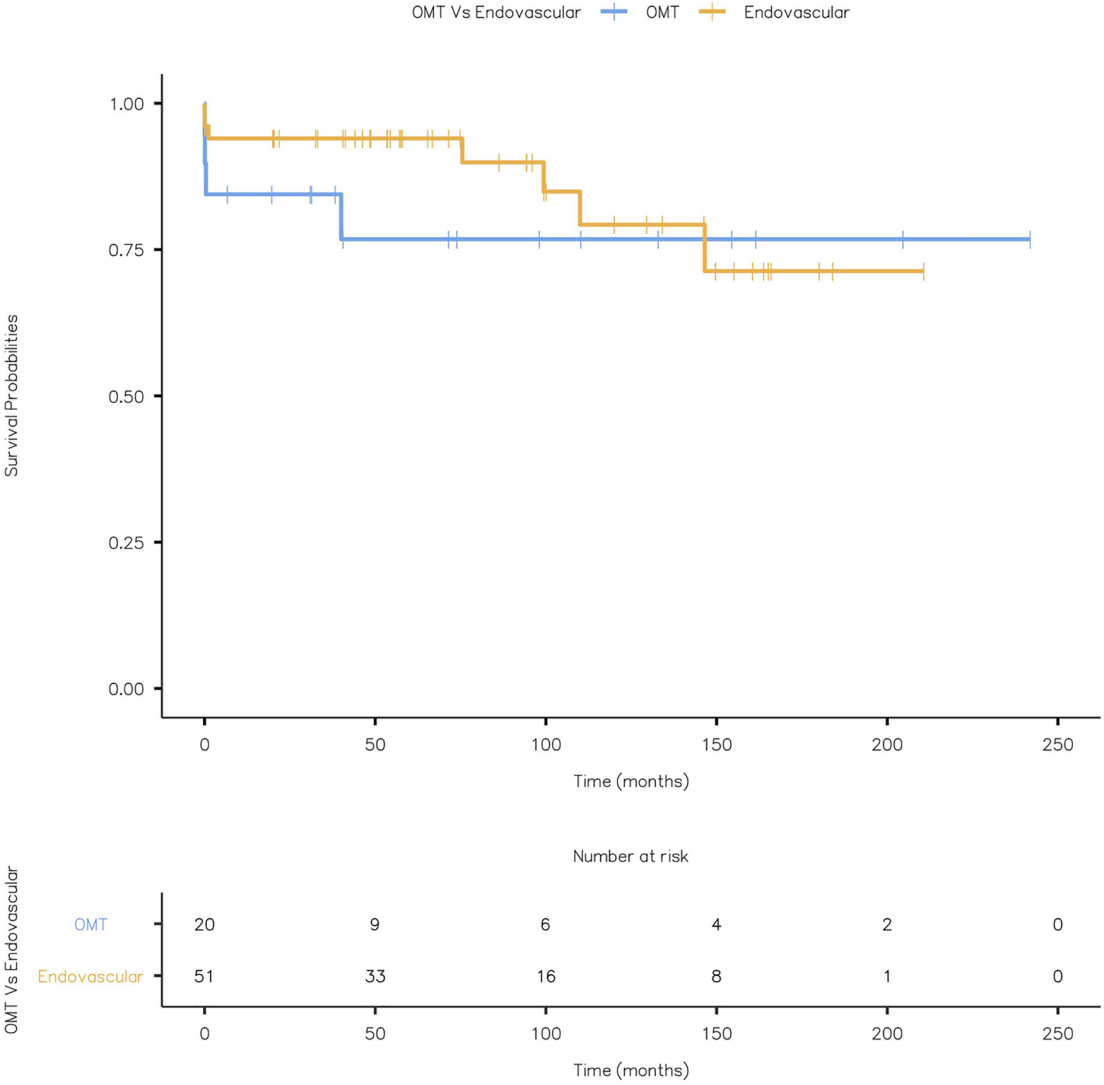
Kaplan Meier Curve displaying combined aneurysm and cardiovascular related mortality (Log-rank test: *X*^2^ = 0.555 and *p* = 0.456).

Four patients with OMT and seven with TEVAR (4 SAD and 3 TAA post-SAD) had combined aneurysm and cardiovascular-related mortality. Kaplan Meier Curve displaying combined aneurysm and cardiovascular-related mortality is given in Figure 14 (Log-rank test: *X*^2^ = 0.555 and *p* = 0.456).

### Post-op complications

Four patients had paraplegia, and all four also developed renal failure; three had undergone TEVAR (1 TAT, 1 SAD and 1 TAA), and one was on OMT for SAD.

**Figure 15:**
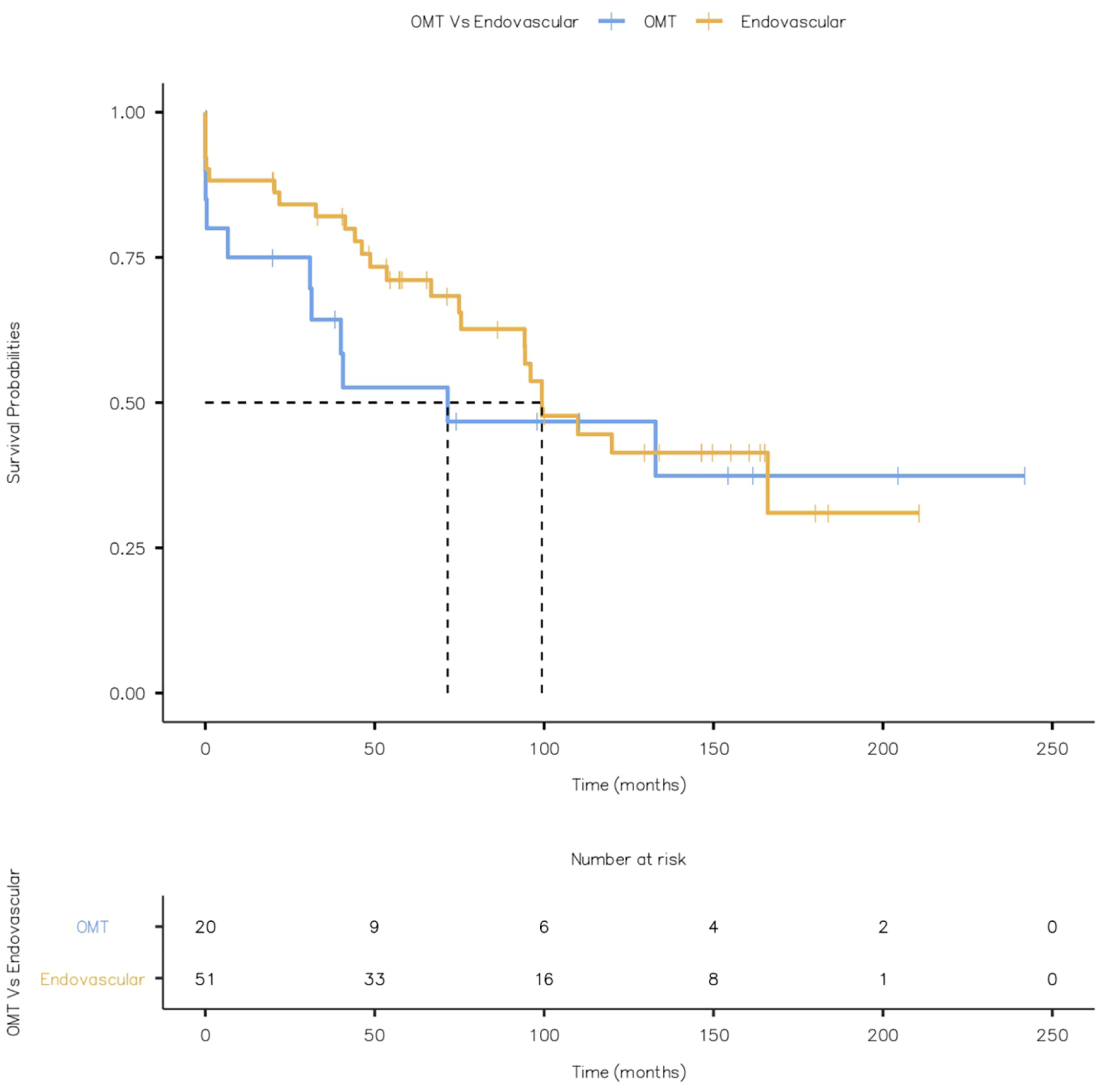
Kaplan Meier Curve displaying all-cause mortality (Log-rank test: *X*^2^ = 0.545 and *p* = 0.460).

None of the patients had a stroke or bowel ischaemia. There was no metachronous malignancy during the follow-up. Kaplan Meier Curve displaying all-cause mortality is given in Figure 15 (Log-rank test: *X*^2 ^= 0.545 and *p* = 0.460).

### Follow-up

The overall average follow-up duration was 78.2 ± 61.6 months.

## Discussion

Acute Aortic Syndromes (AAS), a term first introduced by Vilacosta and Roman in 2001 (1), are a group of interrelated potentially lethal emergency conditions of the aorta (14, 15). Regardless of the exact underlying pathology, the presenting complaint is chest or interscapular pain with imminent life-threatening scenarios. Despite outstanding diagnostic and therapeutic advancement over the past several decades, mortality and morbidity of AAS remain stubbornly high (8, 16).

Penetrating aortic ulcer (PAU) involves a breach of the internal elastic lamina into the media; however, AD and IMH appear mainly in the media, whereas traumatic aortic injury can strike any of the three aortic layers. The consequence of PAU and IMH is unpredictable and fatal in many patients as they induce aortic dissection, aneurysm and/or rupture, as depicted in our study (17).

ESC guidelines recommend TEVAR for complicated type B aortic dissection defined by persistent or recurrent pain, uncontrolled hypertension on OMT, early aortic expansion, mal-perfusion, and signs of rupture, including haemothorax, increasing periaortic and mediastinal haematoma (8, 18). However, TEVAR alone may be contraindicated or unfeasible in several cases and may require hybrid techniques. We avoid full open surgical correction for AAS, as it carries up to 50% risk of in-hospital mortality and major adverse clinical events, such as spinal cord ischaemia, stroke, mesenteric ischaemia infarction, and acute renal failure (8, 18, 19).

An aggressive early endovascular/hybrid treatment in highly selected cases, especially in patients with dynamic/static obstructive mal-perfusion syndrome, must be contemplated, with the optimal mode of intervention based on the comprehensive evaluation of the individual patient's anatomical and physiological status. In the right hands, this will offer an acceptable prospect of survival to AAS patients, who otherwise have a bleak prognosis (8, 11). We staged TIGER or open surgical debranching when the condition and time permitted, to manage the acute organ mal-perfusion, and later we stent the proximal thoracic aorta (11).

In our study, the most common presentation of an AAD patient is a male, heavy smoker in his seventh decade with a history of hypertension who present with insidious onset of interscapular pain or chest pain. All of our patients had an elevated D-dimer. Ruling out AAD on blood results is challenging and not advisable given the almost ubiquitous access to cross-sectional imaging in modern clinical practice; however, we concur with the finding of Suzuki et al*.* (20) that if a D-dimer threshold of 1,600 ng/ml is not reached in the first 6 h of symptom onset, then it is safe to rule out AAD. Consistent with the ESC guidelines, all of our patients with AAD were prescribed beta-blockers when tolerated, and the use of vasodilators was decreased (8, 16).

Adapting endovascular techniques to the thoracic and thoracoabdominal aorta differs from how they have been applied to the abdominal aorta. Firstly, there is an actual increase in the incidence of thoracic aortic pathologies, unlike the abdominal aorta, where the incidence of aneurysms is decreasing (21). Secondly, endovascular techniques, although widely embraced for the descending thoracic aorta, are not yet well developed for the thoracoabdominal aorta, and OSR remains the gold standard in this area (22, 23). Finally, there are a greater variety of pathologies within the thoracic aorta, with disease patterns more likely to include dissection, acute transection, penetrating aortic ulcer, intramural haematoma, coarctation and disease patterns relating to connective tissue disorders (21).

Ten years ago, endoluminal therapy supplemented rather than supplanted open repair, mainly because the only commercially available devices that were FDA-approved in the US treated diseases with isolated descending the thoracic aorta. However, within three years of US Food and Drug Administration (FDA) approval, TEVAR rates across various geographic regions grew exponentially. In certain units, EVAR surpassed AAA repair with minimal variance in utilization across USA and Europe (21). However, the repair of more complex TAA diseases is still confined to centres of excellence. Traditional open repair of these types of aneurysms requires formidable skill and enormous clinical resources driving a concentration on centres of excellence as a cardinal concept in the field (22). In such centres, OSR of the thoracic aorta is associated with good short-term and long-term technical and functional outcomes (22, 24). However, outside of such centres, the results of OSR are disappointingly low. Similarly, recent reports have begun to espouse notions of centres of excellence in the endovascular management of acute aortic syndromes (25, 26).

There is a distinct difference between gender and ethnicity in AAS, where females suffer more from IMH than males, while males carry the brunt of aortic dissection (10). However, IMH has a higher incidence in Asians compared to Americans or Europeans (27). Japanese and Koreans manage IMH initially without surgery, and surgery is reserved for those who acquire complications due to IMH (28, 29). Conversely, in North America, Europe and China, medical management mortality is absurdly high and urgent surgical intervention is always the gold standard (30–33).

In our experience, all AAD cases presenting with visceral or cerebral mal-perfusion bore high mortality. However, this should not discourage immediate operative repair in younger patients (Figures 2–11) (10). Moreover, aortic diameter >50 mm and thickness of the IMH >16 mm are risk factors for death, rupture and translation to AD in patients who are medically treated (28, 29). This was documented in three-quarters of all of our IMH patients who succumbed to aneurysm-related complications.

According to existing recommendations, acute aortic arch dissections are handled surgically. Similarly, real-world clinical experience advocates open surgery in dissections with retrograde extension into the ascending aorta. In contrast, in uncomplicated patients, arch dissections with extension into the descending aorta and no ascending involvement are treated medically or endovascularly (8, 34–36).

In contemporary practice, aortic centres manage AAS with proximal arch entry tear without ascending aorta involvement with medical therapy. These patients are branded as “non-A, non-B aortic dissection” (4) are managed as a type B aortic dissection. We concur with Trimarchi et al*.* (37) that such patients must be dealt with a patient-specific approach.

The concept of grouping this range of clinical conditions with a universal risk to life into a single syndrome makes sound clinical sense. Precedence exists in cardiology whereby the term acute coronary syndrome depicts a number of different clinical presentations of threatened coronary artery occlusion to be considered together to promote early identification, enable clinical treatment pathways and enhance survival (11, 38, 39).

In this study, chimney grafts were used with excellent technical success for subclavian branches. They empirically preserve flow to the left subclavian artery (LSA). However, there are some reports that demonstrate no neurological sequel from coverage of the left subclavian artery during TEVAR. A meta-analysis by Rizvi et al*.* (40) found that LSA coverage increases the risk of arm ischaemia, vertebrobasilar ischaemia, spinal cord ischaemia and anterior circulation stroke. Another meta-analysis by Cooper et al*.* (41) found that pre-emptive revascularisation offered no protection against cerebrovascular accidents, suggesting heterogeneous aetiology. However, they did favour the revascularisation of the LSA for reducing the risk of spinal cord ischaemia. The SVS recommends pre-emptive revascularisation of the LSA in all cases where feasible, and they increase the strength of the recommendation to grade 1 in patients whose anatomy means that coverage of the LSA would compromise the perfusion of critical organs (42). This recommendation applies to patients who have an internal mammary to coronary bypass graft or to patients with contralateral vertebral artery stenosis or occlusion, potentially compromising vertebrobasilar circulation. These recommendations relate to elective circumstances, and the recommendations become weaker in the acute setting. However, all of our patients had left subclavian revascularization by endo or transposition.

Cognisant of the increased risk of spinal cord ischaemia with LSA coverage, concomitant revascularisation was examined. When deciding on the method of revascularisation, there is no strong evidence available to promote one method over the other. However, LSA transposition is associated with potential complications of a stroke, bleeding, and injury to the thoracic duct and damage to neck nerves, including the phrenic, vagus, sympathetic chain and brachial plexus. Chimney stenting is minimally invasive and can be performed simultaneously, which is helpful in an emergency setting; however, we performed eight cases of transposition of the left subclavian artery to the left common carotid artery to increase the proximal landing area without complications. The rationale is if the distance is more than 2 cm to the left common carotid, and the left subclavian chimney will require two long subclavian stents that could encroach on the lumen of the left common carotid artery or even induce retrograde type A dissection.

Although endovascular adjuvants such as chimney grafts have proven effective in the emergent situation, at least in the short-term, they are not always applicable, especially in the treatment of thoracoabdominal pathologies (43). Pre-emptive or staged revascularisation was utilised in this study. We focused our primary approach on the acute aortic presentation and then the secondary staging intervention if required to deal with leftover pathology on an elective basis. Similar to the current study, others have used hybrid repair in the emergent setting for patients who are poor surgical candidates with varying degrees of success (44, 45). Intuitively, since they do not require a thoracotomy, hybrid techniques for the repair of thoracoabdominal aortic pathologies should entail fewer systematic and cardiac complications with less postoperative pain, less blood loss and fewer coagulation disorders, a reduced rate of SCI, reduced duration of mesenteric and visceral ischemia, diminished bacteria translocation and sepsis, and reduced renal failure, all resulting in reduced intensive care unit and hospital stay when compared to open repair (45, 46).

However, meta-analysis demonstrates a 30-day mortality rate of 12.8% (95% CI, 8.6%–17.0%), as high as open repair (45). Furthermore, there is massive heterogeneity in the morbidity and mortality rates among different medical centres. Analysis of the technique's feasibility in itself reveals overall primary technical success exceeds 96%, and midterm visceral graft patency rates are encouraging (96.5%, mean follow-up 34.5 months). Technical difficulties do not explain the substantial mortality associated with the debranching technique. With successful outcomes confined to higher volume centres, this can be attributed to the increased learning curve of the technique and to the early identification and appropriate management of postoperative complications. In fact, the overall renal function impairment requiring haemodialysis remains high, at a pooled rate of 8.8%, and the meta-analysis reported 7.0% spinal cord ischemia symptoms, 1.7% rate of cerebral stroke, 5.2% bowel necrosis, bowel infarction and multiple organ failure were the most common causes of 30-day/in-hospital death (45). The staged hybrid procedure seems to be associated with a decreased rate of renal insufficiency but with an increased possibility of aneurysm rupture (11, 47). This complication occurred in our series and is all the more pertinent in the scenario of ASS, where the risk of aortic rupture is already high.

Branched stent grafts have emerged as a conceptually less invasive yet technically demanding alternative for the treatment of thoracoabdominal pathologies with mixed results (48). The need for customisation of these devices to fit individual patient anatomies used to demand a six-week wait, and accordingly made these devices impossible to use within the acute setting. However, the current availability of standardized devices has broadened the indications for use, albeit still within some anatomical constraints (49). Haulon et al*.* (49) reported a 9% in-hospital mortality rate among 33 patients unfit for open surgical repair treated with this technique and a transient spinal cord ischemia in 12% and permanent paraplegia in 3%. Intermediate-term patency and survival with this technique seem hopeful; however, mortality and spinal cord ischemia risks are still considerable (48, 49). The main concern that prohibits the use of these devices in the acute setting is not their limited availability or even their considerable cost, and rather it is the profound systemic inflammatory reaction that patients are subjected to post-implantation. Leukocytosis, thrombocytopenia and renal injury are uniform following fenestrated and branched interventions, with the severity of the inflammation and coagulopathy correlating with the degree of renal injury (50). In the context of ASS, such an intense systemic reaction is all the more difficult for these patients to tolerate.

Our four paraplegic patients developed renal failure and died. All of them had TAA post-SAD. All mortalities occurred after seven days when the patient was transferred to the referring hospital. Bouts of hypotension with low haemoglobin contributed to these catastrophic events. Intensivists must understand that the indications to maintain haemoglobin level post-TAA repair differ from their criteria for blood transfusion. In addition, adjuvant measures such as preservation of the perfusion of the left subclavian artery are extremely important (51).

AAS patients with concomitant type A dissection have an acute unoperated mortality rate of 2% per hour (52). Timely delay in diagnosis and referral continues to hinder definitive diagnosis and management. Key starter role (16) of peripheral units must have an accurate triaging capability with the ability to utilise targeted clinical risk scores until the patient is transferred to a centre of excellence. All AAS patients, regardless of the initial therapeutic strategy, whether medical, interventional, or surgical, have the entire aorta and its branches unstable and must be considered for lifelong surveillance. All AAS patients are at high risk for re-dissection, aneurysm formation, and rupture even after the acute index event's efficacious therapy (53). In order to prevent the progression of IMH to dissection, or rupture, we have a policy of early intervention on IMH as our experience mirrors that of Mesar et al*.* (54).

Acute and subacute patients have more complications at presentation, whereas chronic patients display more aneurysmal dilatation and actual lumen collapse. Our experience has taught us that the dissecting aortic membrane is fragile in the acute phase, which results in distal erosions and retrograde type A dissection. However, patients with chronic type B aortic dissection (CTBAD) had a less compliant membrane that hindered aortic remodelling (11, 40–42, 46–48, 55, 56).

We employ the “Time Until Treatment Equipoise” TUTE strategy (57), which is the point in time during follow-up after which an intervention is most beneficial because the mortality risk of intervention is lower than the mortality risk of continuing current medical management (11, 40–42, 46–48, 55, 56).

We offer endovascular scissoring, kinetic elephant trunk and TIGER techniques for chronic symptomatic aortic dissection patients to eradicate the fibrous rigidity of the intimal flap and create a single aortic lumen. By abolishing the multiple distal re-entry tears in the subdiaphragmatic aorta, the whole aorta can be stabilized and blood flow laminated through all visceral branches (11, 40–42, 46–48, 55, 56).

Predictors of worse outcomes and indications for intervention in acute type b aortic dissection (ATBAD) are the total aortic diameter of >40 mm in the acute phase, patent FL diameter of >22 mm in upper descending TA, partial thrombosis of the false lumen, elliptic TL completely surrounded by rounded FL, periaortic hematoma with unilateral pleural effusion, which is an especially ominous sign of impending rupture, age greater than 55, and visceral mal-perfusion (11, 40–42, 46–48, 55, 56).

In the absence of complications, maintaining an initial reticence to the use of stent-grafts in the acute phase likely leads to a better clinical prognosis after TEVAR and respects the TUTE principle. We agree with previous recommendations that TUTE at 15–90 days is optimal for patients who do not need emergent interventions (11, 40–42, 46–48, 55, 56).

The role of TEVAR for chronic dissection is becoming less controversial, but it is often associated with higher rates of reinterventions, unfavourable aortic remodelling, and long-term major adverse clinical events (49, 50, 58). Our results depict a 7% re-intervention rate over 20 years.

The most catastrophic complication of AAS, particularly IMH and ATBAD, is the 21% incidence of aneurysm rupture (59). Our experience mirrors others of failed medical treatment in IMH with higher mortality than expected. Our in-hospital mortality of managing AAS of 15.5% mirrors the 16.7% reported by Faure et al*.* (60).

When TEVAR fails in resolving end-organ ischaemia due to the persistence of visceral mal-perfusion, even after actual lumen expansion due to static visceral obstruction, this warrants immediate visceral stenting or a bypass because even though trials of endovascular fenestration may succeed in the short term, they have with questionable short to mid-term durability (61).

PETTICOAT and STABLE approaches were designed to deal with malperfusion after TBAD (62–65). However, their widespread use is not justified, as there is not robust enough data about their longer-term benefit, especially in terms of the risk of persistent mal-perfusion of visceral vessels. Instead, they should be used as adjunctive bailout tools in cases complicated by dynamic malperfusion.

In all of our patients, we utilised a personalised approach to prevent mal-perfusion syndrome or visceral ischemia, aided by the ability to perform the hybrid endovascular repair (HER) and TIGER in complex static and dynamic visceral challenges. We had no visceral ischaemia, and our results contradict the finding of Ryan C et al*.* (66), who had 21% visceral ischaemia, which substantially increased their risk of early death by 4.6 fold.

Ten per cent of all AAS develop mesenteric mal-perfusion, which has an in-hospital mortality of 31% (67). This is due to static or dynamic compression, or a combination of static and dynamic compression of the visceral arteries. We believe the TIGER approach is the best way to deal with the combined type, as demonstrated in Figures 2–6.

The rate of spinal cord ischemia and cerebrovascular events in our population was 5.7%, comparable to Eid-Lidt et al*.* (68) but better than other studies, which reported up to 9.4% (69). We have a firm policy of revascularisation of the left subclavian artery and maintaining perfusion to spinal arteries and lumbar's by TIGER technique plus protection of both internal iliac arteries.

Our findings mimic the SVS/STS that PAU rupture risk always correlates to ulcer depth, and PAU with IMH is a risk of aortic rupture and has a worse clinical course than PAU without IMH (70, 71).

Our findings mirror Li et al*.* (72) findings that patients with type B PAU-IMH should be managed more aggressively with endovascular intervention as medical management often fails and results in aortic adverse events, in particular, micro bleeds of the perforated aortic ulcer in the intramural hematoma of the descending thoracic aorta.

We did not witness variations in the outcome of the incidence of ASS between different socioeconomic classes. In total, 65% of our AAS patients had private insurance, and although we would assume selection bias due to insured patients having the privilege of faster referral and access to earlier diagnosis and definitive care, all our patients had similar outcomes because they all received risk factor modification regardless of insurance status (73).

Complicated AAS management requires a precision personalised approach. A policy of mix and match is imperative for a better outcome as there is not one single approach, which can be standardised for all patients.

We forcibly advocate that all AAS must be triaged to designated specific aortic centres to reduce mortality (74). We agree with Davies et al*.* (25) and Harris et al*.* (75) that setting up an acute aortic treatment centre will increase referral volume with prompt diagnosis through a standardized protocol and reduce the time to definitive treatment for AAS patients with improved survival.

Implementation of diagnostic aortic protocols, regionalization of services, and inter-hospital coordination are crucial factors in the care of AAS patients (25, 76, 77). Patients treated in high-volume centres or by high-volume surgeons with a dedicated on-call aortic team will establish a significant reduction in mortality. Pooled adjusted estimates for both high-volume centres and surgeons confirm these survival benefits (74).

Our experience mirrors Weiss et al. (78) finding that AD/IMH/PAU patients have a significant risk of aortic death, any aortic event, and a first-time diagnosis of aortic aneurysm that endures after the acute phase of 14 days from diagnosis. Especially those that survive the initial event without surgery remain at a 10-fold higher risk of subsequent aortic intervention. Kret et al. (79) and Chou et al. (80) reported up to 43% of IMH and over 30% of PAU patients needed delayed intervention. Conversely, traumatic aortic transection patients have reasonably good long-term aortic outcomes.

Weiss et al. (78) validated that, after excluding patients who developed aneurysm formation within 14 days, the risk of developing any first-time thoracic aortic aneurysm is 6.2-fold and an abdominal aortic aneurysm is 2.8-fold higher than in the average population.

Advances in post-diagnosis treatment are indispensable to further the patient's prognosis. In our case, severe AD with TAA and PAU accounted for all subsequent redo-interventions. We realized that AD/IMH/PAU's long-term prognosis is poor and certainly does not match normal population levels.

Of patients who received OMT for uncomplicated TBAD (81), 33% will suffer an aortic event in the first five years. This risk is amplified if aortic growth diameter is recognized within the first six months, with a higher risk of rupture, mal-perfusion, or late aneurysm formation in 15% of the cases. Patients will also remain at risk during later years.

In patients with non-resolving IMH (82), most adverse events are witnessed in the first year and there is about 60% risk of adverse events. Our findings depict the findings of Isselbacher et al*.* (83) that signs of high-risk IMH that could progress to pan aortic dissection and rupture are hematoma thickness >10–13 mm, maximum aortic diameter 45–50 mm, and ulcer projection into the convexity of the aortic arch. A retrospective calculation of SPAU2 score [S: relatively low SBP on arrival, P: presence of pericardial effusion on admission CT, A: relatively large ascending aorta diameter on admission, U2: presence of ulcer-like projection (ULP) on admission CT] for our IMH showed that they lie between 1 and 3 (84). Our policy for IMH is a low threshold for intervention, and we intervene if the SPAU2 score is more than one. Furthermore, a high SPAU2 score (>3) requires immediate intervention. However, IMH with early precipitous aortic growth is a strong gauge for an unfavourable outcome and must be judged as a warning to execute elective intervention.

All of our blunt aortic trauma patients had their intervention within the first 24 h as their Radiographic Severe Injury” (RSI) (81) scores were high. Our patient's management could not be delayed for more than 24 h as they were actively bleeding (grade III and IV), which contradicts the recommendation by Alarhayem et al. (85) that deferring endovascular repair beyond 24 h after injury should be contemplated.

CT scan findings and RSI score indicative of high mortality correlated with Blunt Aortic Injury in the presence of total/partial aortic transection, active contrast extravasation, or the association of 2 or more of the following: contained contrast extravasation >10 mm, periaortic hematoma, and/or mediastinal hematoma with thickness >10 mm, or significant left pleural effusion.

This study is a single surgeon experience, and the series includes variations in the underlying pathology and the modality of treatment used. However, the series is reflective of a modern high-volume vascular centre, and the result adds to the mounting evidence for successful management of these related pathologies under the umbrella of acute aortic syndromes, using progressively TEVAR/TIGER and hybrid endovascular repair, which all are less-invasive techniques than complete open repair. Moreover, this real-world longitudinal study witnessed the chronological shift towards personalised precision medicine to manage AAS. We believe that the safest and most effective therapy in an acute setting is a staged hybrid approach. Grouping acute aortic pathologies into a single syndrome with definite treatment pathway is fundamental for efficient, cost-effective and life-saving therapy. Furthermore, operative treatment plays a crucial but limited role in the overall chance of survival; AAS patients should be managed in acute aneurysm centres with the appropriate high-quality specialist resources.

## Data Availability

The raw data supporting the conclusions of this article will be made available by the authors, without undue reservation.
